# How Charge and Triple Size-Selective Membrane Separation of Peptides from Salmon Protein Hydrolysate Orientate their Biological Response on Glucose Uptake

**DOI:** 10.3390/ijms20081939

**Published:** 2019-04-20

**Authors:** Loïc Henaux, Jacinthe Thibodeau, Geneviève Pilon, Tom Gill, André Marette, Laurent Bazinet

**Affiliations:** 1 Department of Food Sciences and Laboratory of Food Processing and Electromembrane Processes (LTAPEM), Université Laval, Quebec, QC G1V 0A6, Canada; loic.henaux.1@ulaval.ca (L.H.); jacinthe.thibodeau.1@ulaval.ca (J.T.); 2 Institute of Nutrition and Functional Foods (INAF), Université Laval, Quebec, QC G1V 0A6, Canada; Genevieve.Pilon@criucpq.ulaval.ca (G.P.); Andre.Marette@criucpq.ulaval.ca (A.M.); 3 Québec Heart and Lung Institute, Université Laval, Department of Medicine, Quebec, QC G1V 4G5, Canada; 4 Department of Process Engineering and Applied Science, Dalhousie University, P.O. Box 15,000, Halifax, NS B3H 4R2, Canada; Tom.Gill@Dal.Ca

**Keywords:** electrodialysis with filtration membrane (EDFM), triple size-selective separation, glucose uptake, bioassay-guided validation, bioactive peptides

## Abstract

The valorization of by-products from natural organic sources is an international priority to respond to environmental and economic challenges. In this context, electrodialysis with filtration membrane (EDFM), a green and ultra-selective process, was used to separate peptides from salmon frame protein hydrolysate. For the first time, the simultaneous separation of peptides by three ultrafiltration membranes of different molecular-weight exclusion limits (50, 20, and 5 kDa) stacked in an electrodialysis system, allowed for the generation of specific cationic and anionic fractions with different molecular weight profiles and bioactivity responses. Significant decreases in peptide recovery, yield, and molecular weight (MW) range were observed in the recovery compartments depending on whether peptides had to cross one, two, or three ultrafiltration membranes. Moreover, the Cationic Recovery Compartment 1 fraction demonstrated the highest increase (42%) in glucose uptake on L6 muscle cells. While, in the anionic configuration, both Anionic Recovery Compartment 2 and Anionic Recovery Compartment 3 fractions presented a glucose uptake response in basal condition similar to the insulin control. Furthermore, Cationic Recovery Compartment 3 was found to contain inhibitory peptides. Finally, LC-MS analyses of the bioassay-guided bioactive fractions allowed us to identify 11 peptides from salmon by-products that are potentially responsible for the glucose uptake improvement.

## 1. Introduction

Type 2 diabetes (T2D) is a complex multifactorial disorder resulting from insulin resistance in peripheral tissues, such as skeletal muscle, and pancreatic β-cell dysfunction [1]. This disease is growing at an alarming rate and is predicted to account for more than 350 million cases by 2030 [2]. Skeletal muscle is the major site of glucose uptake in the postprandial state and the development of insulin resistance in this tissue is considered a cornerstone in the pathogenesis of T2D. Interestingly, increased fish consumption has been suggested to protect against metabolic syndrome (MetS), type 2 diabetes and cardiovascular disease (CVD) in obese subjects [3,4]. Our group previously showed that fish protein is an important contributor to these beneficial effects. Indeed, Pilon et al. (2011) showed that salmon protein hydrolysate reduced inflammation in visceral adipose tissue and improved insulin sensitivity in an animal model of diet-induced obesity [5]. Furthermore, we recently reported that in a mouse model of obesity, a low molecular weight peptide (LMWP) fraction (<1 kDa) from the proteolytic digestion of salmon filleting waste improved glucose tolerance and lipid homeostasis [6]. Interestingly, other fish protein sources than salmon have been reported for their metabolic properties in animal models and also in humans [7,8,9,10,11,12,13]. Therefore, proteins from marine by-products definitively represent a high potential for the development of functional foods and nutraceutical products [7,8,9,10,11,12,13]. On the other hand, the value-added aspects of organic by-products have become a priority in order to respond to the sustainability, environmental, economic, and regulatory challenges [14,15].

Consequently, production, separation, and characterization of bioactive peptide (BP) by-products are important issues for the food and biopharmaceutical industries, and we are now aware that BPs’ activity depends on specific molecular and chemical properties [16]. For example, the surface charge of peptides has been shown to be an important factor for the expression of their bioactivity, and they have to be selectively recovered to maximize their activity [17]. However, since enzymatic hydrolysis is used to liberate BPs from the protein matrix [18], generating complex peptide mixtures, a sustainable technique allowing for the selective purification and concentration of these BPs or peptide fractions from complex mixtures is needed. Also, it was demonstrated that BPs may have low molecular weight depending on the conditions of digestion and the types of proteolytic enzymes used [19,20]. Amongst the available technologies for peptide separation, pressure-driven processes such as ultrafiltration, nanofiltration [21,22,23], and chromatographic methods [24,25] are perhaps most frequently used. However, pressure-driven techniques sometimes fail to separate molecules of similar size and are susceptible to membrane fouling [26]. Chromatography is too costly, slow, not applicable for the fractionation of large sample volumes, and sometimes uses organic solvents [27]. Isoelectric focusing is perhaps a more biocompatible separation technology that is most often used on a laboratory scale and, more recently, on a larger scale, but as mentioned by Hashimoto et al. (2005, 2006) [28,29], the limiting volume (50 L), the degradation of agarose gels after prolonged time (8 h) of peptide fractionation, and high voltages (500–600 V) led researchers toward alternative methods for an optimal separation and purification. More recently, electrodialysis with filtration membrane (EDFM), a “green” and ultra-selective process, was developed for separation/concentration of bioactive ingredients. EDFM is based on the size exclusion capabilities of porous membranes with the charge selectivity of electrodialysis. In comparison with other common technologies used for biomolecule separation, EDFM has many unique advantages: it is environmentally-friendly, using no organic solvents or dangerous chemicals; it is highly selective for targeted molecules; it operates at low pressure and therefore reduces membrane fouling; it allows simultaneous molecular separation and concentration as well as preservation of the feed solution’s commercial value. Recently, Roblet et al. (2016) used the EDFM process to fractionate a low molecular weight (<1 kDa) salmon protein hydrolysate and demonstrated that the basal glucose uptake as well as insulin-stimulated glucose uptake were enhanced by 40% and 31%, respectively, at pH 6 in the final feed compartment [30]. However, in that work, only one molecular weight cut-off (MWCO) of 20 kDa was used for the ultrafiltration (UF) membrane and consequently no discrimination according to the peptide size between recovery fractions and their glucose uptake response was possible.

Hence, in a context of eco-efficiency and to create value-added products, the objectives of the present study were (1) to simultaneously separate specific peptide fractions, according to their charges as well as MW, from a salmon protein hydrolysate by EDFM by stacking three UF membranes of different molecular weight exclusion limits (50, 20, and 5 kDa), (2) to characterize the peptide fractions obtained after separation in terms of molecular weight profiles and sequences, and (3) to measure in vitro the level of glucose uptake response of these fractions in the presence or absence of insulin stimulation in L6 skeletal muscle cells, following this charge and size separation.

## 2. Results and Discussion

### 2.1. Evolution of Peptide Concentration and Final Migration Rates

**Figure 1:**
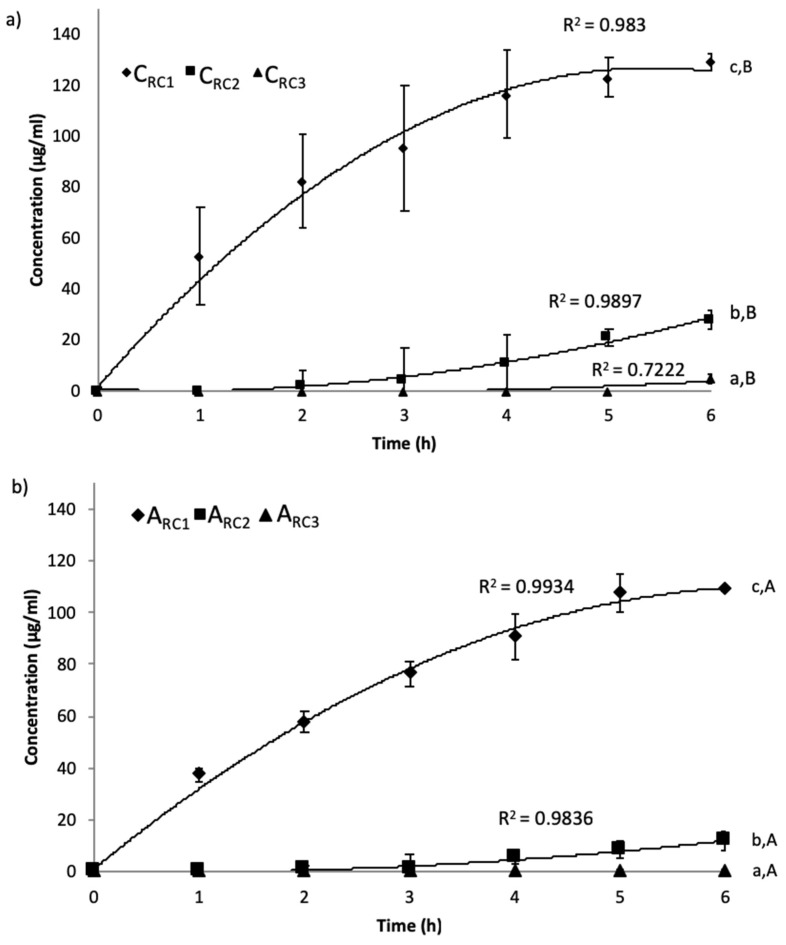
Evolution of peptide concentration in (**a**) cationic (Cationic Recovered Compartment 1, Cationic Recovered Compartment 2, Cationic Recovered Compartment 3, respectively, C_RC1,_ C_RC2_, and C_RC3_) and (**b**) anionic (Anionic Recovered Compartment 1, Anionic Recovered Compartment 2, Anionic Recovered Compartment 3, respectively A_RC1_, A_RC2_, and A_RC3_) compartments during 6 h of the electrodialysis with ultrafiltration membrane (EDUF) process. Lowercase letters are used to compare the three recovered compartments of the same configuration where capital letters are used to compare the recovered compartments between anionic and cationic configurations. Values followed by different letters were statistically different.

The patterns of peptide separation and concentration as a function of time in recovery compartments of both cationic and anionic configurations measured by the micro-BCA method are represented in Figure 1.

As expected, significant differences (*p* < 0.05) were obtained concerning the peptide concentrations in the recovery compartments in the order of C_RC1_ > C_RC2_ > C_RC3_ for the cationic configuration and A_RC1_ > A_RC2_ > A_RC3_ for the anionic configuration. Indeed, final concentrations obtained for cationic fractions were 129.10 ± 3.57; 27.74 ± 3.61 and 4.78 ± 1.80µg/mL corresponding to peptide migration rates (Table 1) ranging between 0.13 and 3.9 g/m^2^·h. At the same time, the final concentrations recovered in the anionic configuration were 108.91 ± 0.41µg/mL and 11.51 ± 3.66µg/mL, corresponding to peptide migration rates between 0.24 and 2.24 g/m^2^·h (Table 1). No significant peptide migration (*p* > 0.05) was observed in the A_RC3_ compartment after 6 h of electrodialysis with ultrafiltration membrane (EDUF) treatment. However, in this particular sample, nitrogen content analysis by LECO (Table 1) showed the presence of very low concentrations of peptides (0.15%) (p/p) in the final powder, after lyophilisation of the recovery compartments.

Peptides have to migrate further from the inlet feed solution to reach compartment 2 and even further to reach compartment 3 as compared to compartment 1. So, only low molecular weight peptides and/or larger peptides with high charge density could reach compartments 2 and 3 and migrate through the UF membrane with a MWCO of 20 kDa (UF-20 kDa) and the UF membrane with a MWCO of 5 kDa (UF-5 kDa). This was confirmed by previous studies carried out on flaxseed cationic peptides [31] and snow crab anionic peptides [32] for a configuration composed of two UF membranes with different MWCOs. Differences observed between the cationic configuration and the anionic configuration could be due to the higher cationic peptide concentrations generated by the successive digestion with pepsin and trypsin/chymotrypsin. These results were in accordance with work by Udenigwe et al., (2012) [33] on flaxseed hydrolysate protein, where a higher peptide concentration was observed in the cationic compartment after the EDUF separation. Moreover, in the present study, for the C_RC1_ and A_RC1_ compartments, results showed a linear increase of the migration rate during the first four hours of EDUF treatment, and then a slowdown appeared in migration rates during the last two hours. These results could be due to an alteration of the membrane (UF membranes and ion-exchange membranes (IEMs)) integrity or to a membrane fouling. Indeed, the thickness and conductivity of each membrane of both configurations were determined before and after three repetitions. No differences were observed concerning the thickness whatever the membrane for both configurations. Nevertheless, a decrease of the conductivity of the UF membranes and the IEMs for both configurations could indicate an internal and irreversible fouling by peptides or free amino acids. Indeed, Suwal et al. (2015) have observed an irreversible fouling by free amino acids in internal nano-pores of IEM during EDUF separation [34].

### 2.2. Characterization of Peptide Profile by RP-UPLC-MS

**Figure 2:**
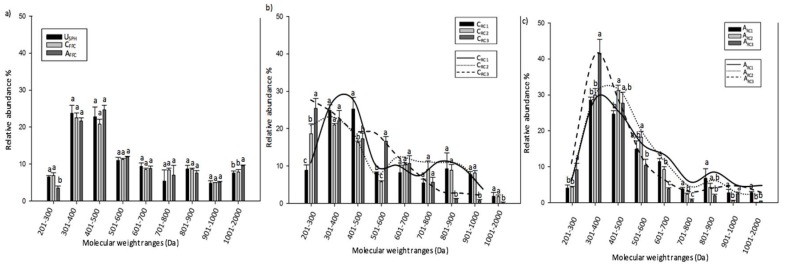
Profiles of peptide molecular weight in (**a**) U_SPH_, C_FFC_, and A_FFC_, (**b**) cationic compartments (C**_RC1_,** C**_RC2_**, and C**_RC3_**) and (**c**) anionic compartment (A_RC1_, A_RC2_, and A_RC3_) generated after 6 h of the EDUF process. Means with different lowercase letters within a molecular weight range are significantly different (*p* < 0.05).

Peptides of low molecular weight (MW) in the 301–500 Da range were the most prevalent in the unfractionated salmon protein hydrolysate (U_SPH_) (46.6% of total abundance) (Figure 2a).

These results were in accordance with previous results obtained by Roblet et al. (2016) [30]. Indeed, similar relative abundances were found for molecular weight ranging from 301 to 500 Da. After 360 min of EDUF treatment, a significant decrease (*p* < 0.005) was observed for levels of peptides ranging from 201 to 300 Da in the Anionic Final Feed Compartment (A_FFC_) and a significant increase (*p* < 0.05) for levels of peptides from 1001 to 2000 in the anionic collection compartments (A_FFC_). No difference (*p* < 0.05) was observed between amounts of peptides accumulated in the Cationic Final Feed Compartment (C_FFC_) and A_FFC_ compartments for the peptides from other size ranges (Figure 2a).

Figure 2b compares the abundance of the different peptides in terms of MW after 6 h of EDUF separation among the C_RC1_, C_RC2_, and C_RC3_ compartments. It appeared that the peptide abundances followed a normal distribution that was shifted toward the low molecular weight peptides (LMWPs) as the migration progressed. Indeed, maximal accumulation of peptides for the C_RC1_ (25.26 ± 3.08%) was observed for MWs ranging from 401 to 500 Da. The C_RC2_ maximal abundance of 20.96 ± 0.38% was observed for MWs ranging from 301 to 400 Da while for C_RC3_, highest peptide accumulations (25.40 ± 2.69%) was observed for MWs ranging from 201 to 300 Da_._

The peptide abundances obtained from the anionic configuration are shown in Figure 2c. Concerning anionic recovery compartments, the majority of peptides ranged in size from 301 to 500 Da. The results also demonstrated that A_RC3_ contained the highest peptide accumulations for MWs ranging from 201 to 300 Da and 301 to 400 Da (9.19 ± 1.82 and 41.64 ± 3.79% of the total accumulation, respectively) compared to A_RC2_ and A_RC1_. Indeed, due to their highest charge and/or lower MWs, peptides ranging from 201 to 400 Da were more able to cross all UF membranes and reach the last compartment. Peptides with MWs between 401 to 500 Da and 501 to 600 Da were significantly higher in the A_RC2_ compared to the A_RC1_ and A_RC3_, respectively. Finally, the level of high molecular weight peptides (HMWPs) (over 601 Da) was higher in the A_RC1_ (27.69 ± 1.85%) compared to the A_RC2_ and A_RC3_ (16.33 ± 1.58 and 9.40 ± 0.51%).

As expected, a decrease in the average size of peptides was observed as follows: C_FFC_ > C_RC1_> C_RC2_> C_RC3_ for the cationic configuration and A_FFC_ > A_RC1_ > A_RC2_ > A_RC3_ for the anionic configuration, which confirmed the high selectivity of the EDUF process.

### 2.3. Relative Energy Consumption

The relative amount of energy consumed is a measurement of the energy used for the migration of one gram of peptides and is reported in Table 1. The results were found to be 512 and 849 Wh/g for cationic and anionic configurations, respectively. The lowest energy consumption was observed for the cationic configuration due to its higher global migration rate. Indeed, as previously demonstrated by Koumfieg Noudou et al., (2016), the increase of the inlet peptide concentration resulted in a decrease of the relative energy consumption [35]. The relative energy consumption varied depending on the cell configuration, the voltage applied, and the peptide migration rate, as demonstrated in previous works, with values ranging from 3.53 to 631 Wh/g [32,35].

### 2.4. Glucose Uptake Experiments

**Figure 3:**
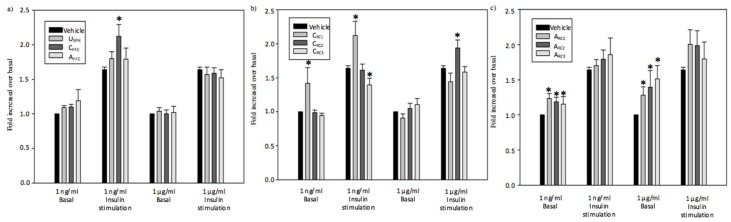
Glucose uptake modulation in L6 skeletal muscle cells in absence or presence of insulin stimulation by (**a**) U_SPH_, C_FFC_, and A_FFC_, (**b**) cationic compartments (C_RC1_, C_RC2_, and C_RC3_) and (**c**) anionic compartments (A_RC1_, A_RC2_, and A_RC3_) generated after 6 h of the EDUF process. One asterisk indicate that mean values are significantly different (*p* < 0.05) than the mean value for the control.

The effects of salmon peptide (recovered, initial, and post treatment) fractions on in-vitro glucose uptake on L6 skeletal muscle cells using two different peptide concentrations (1 ng/mL and 1 µg/mL) were measured in basal and insulin-stimulated conditions. Results presented in Figure 3a show a significant enhancement (*p* < 0.05) of insulin-stimulated glucose uptake for the C_FFC_ at 1 ng/mL (29%) but not for the U_SPH_ and A_FFC_ (in absence or presence of insulin stimulation). However, glucose uptake was not affected by any of the fractions used (U_SPH_, C_FFC_, or A_FFC_) at 1 µg/mL in the presence or absence of insulin. These results are in accordance with previous works of Roblet et al. (2016) [30], where a limited effect of the initial salmon protein hydrolysate was observed at 1 ng/mL and 1 µg/mL. While the final solution recovered in the feed compartment showed a significant enhancement of the glucose uptake at pH 6 [30].

As shown in Figure 3b, after 6 h of EDUF separation in the cationic configuration, only C_RC1_ showed a significant bioactivity (*p* < 0.05) at 1 ng/mL on both basal (42%) and insulin-stimulated glucose uptake (29%) but not at 1 µg/mL. Conversely, C_RC2_ significantly enhanced glucose uptake (18%) in the presence of insulin at 1 µg/mL but not at the lower concentration (Figure 3b). Interestingly, C_RC3_ was found to significantly decrease (15%) insulin-induced glucose uptake (*p* < 0.05) at 1 ng/mL but had no effect at the higher concentration or on basal glucose uptake. According to these results, it appeared that the C_RC3_ fraction has no anti-diabetic potential, suggesting that the C_RC3_ may contain some inhibitory peptides. The C_FFC_ fraction, obtained from the U_SPH_ at the end of the EDUF process, and thus depleted in cationic peptides, was found to have higher glucose uptake activity than U_SPH_ in the presence of insulin. In addition, C_RC1_ presented a similar or higher glucose uptake response than C_FFC_ independent of the condition. It is also important to note that, in the basal state, the C_RC1_ at 1 ng/mL was able to stimulate glucose uptake to the same extent as insulin alone. Additionally, at 1 µg/mL, since no significant increase in glucose uptake for both C_FFC_ and C_RC1_ was reported in the presence or absence of insulin stimulation, let us conclude that their glucose uptake response was not dose-dependent, suggesting that some neutral peptides in the fractions may have masked the bioactivity of the positive ones. Concerning the glucose uptake stimulation in the basal condition, these results were in accordance with previous works obtained by Roblet et al. (2016) [30]. Those authors demonstrated a significant enhancement of the glucose uptake in the absence of insulin stimulation for the cationic fraction at pH 6 as the C_RC1_ [30]. Difference appeared for the bioactivity in the presence of insulin stimulation. Indeed, in previous works, the glucose uptake was not affected by the cationic fraction while C_RC1_ and C_RC2_ showed a significant enhancement of the glucose uptake with insulin stimulation. These differences could be due to the EDUF configuration (three UF membranes with MWCOs of 50, 20, and 5 kDa vs. one UF membrane with a MWCO of 20 kDa) and separation parameters (duration: 6 h vs 1 h; electric field strength of 6 V/cm vs, 14 V/cm; initial peptide concentration of 0.7% vs. 2% [30]) which allowed for the recovery of a higher peptide concentration and a higher diversity of peptides. Moreover, amongst all cationic peptides separated in the different fractions, using Mass Profiler Professional software, MWs and retention time (Table 2) of seventeen peptides were found to be simultaneously and specifically present in all three bioactive fractions (C_FFC_, C_RC1_, and C_RC2_). Thereafter using the Spectrum Mill MS Proteomics software and a specific protein Salmo salar database from NCBI [36], five peptides were identified; their sequences, net charges, and protein precursors are not shown here due to confidential issues (a patent application is in progress).

Concerning anionic fractions, all recovered fractions (A_RC1,_ A_RC2_, and A_RC3_) demonstrated a significant enhancement of the bioactivity (*p* < 0.05) for both concentrations (1 ng/mL and 1 µg/mL) at the basal level (Figure 3c) and a tendency (not statistically significant, *p* > 0.05) to be increased in insulin-stimulated conditions. Moreover, very interestingly, both A_RC2_ and A_RC3_ showed the same increase in glucose uptake (*p* = 0.31 and *p* = 0.55, respectively) compared with that of insulin, while A_RC1_ was not able to reach the same level of bioactivity (*p* = 0.01). That could be explained by the selectivity of the process leading to the concentration of bioactive peptides in the second and last compartments. Nevertheless, the A_FFC_ fraction that was depleted in anionic peptides did not show any improvement of glucose uptake. Roblet et al. (2016) [30] also observed a significant effect of anionic fractions recovered at both pH 3 and pH 9 on glucose uptake modulation in the absence of insulin stimulation, while a limited effect was observed for the anionic fraction obtained at pH 6 [30]. The greater effect obtained on glucose uptake modulation by the three A_RC_ (A_RC1,_ A_RC2_, and A_RC3_) fractions compared to the anionic fraction obtained in previous work could be explained, as for the cationic fractions, by differences concerning the EDFM configuration and separation parameters [32,37]. Using the same method as for cationic peptides, the MWs of twenty-one anionic peptides present in all bioactive anionic recovered fractions (A_RC1_, A_RC2_, and A_RC3_) were identified (Table 2). Amongst these twenty-one peptides, six peptides were confirmed and characterized. As mentioned previously for cationic peptides, the characteristics and sequences of anionic peptides are not shown here due to an in-progress patent application.

Finally, anionic peptides increased glucose uptake in the absence of insulin stimulation, while cationic peptides increased it in the presence of insulin stimulation. In skeletal muscle cells, glucose uptake can be modulated by at least two different signaling pathways: IRS-1/PI3K/Akt (insulin dependent) and 5′-AMP-activated protein kinase (AMPK) (insulin independent) [38]. As previously explained, anionic peptides seem to stimulate the glucose uptake in the absence of insulin stimulation, while for cationic peptides, a better response was obtained in the insulin-stimulation condition; it is possible that the anionic and cationic peptides reported in Table 2 stimulate different pathways involved in glucose uptake. These two pathways are well known for their critical role for glucose transporter translocation to the muscle cell surface in the presence or absence of insulin. Moreover, these pathways were identified as therapeutic targets of anti-diabetic drugs such as metformin and thiazolidenediones, the activation of these pathways by EDUF-isolated salmon bioactive peptides could represent a therapeutic or preventive potential of T2D [39]. To verify this hypothesis, further investigation should be carried out to confirm if these SPH peptide fractions are potential activators of the IRS-1/PI3K/AKT and/or AMPK pathways.

## 3. Materials and Methods

### 3.1. Materials and Electrodialysis Cell

#### 3.1.1. Electrodialysis Configurations

**Figure 4:**
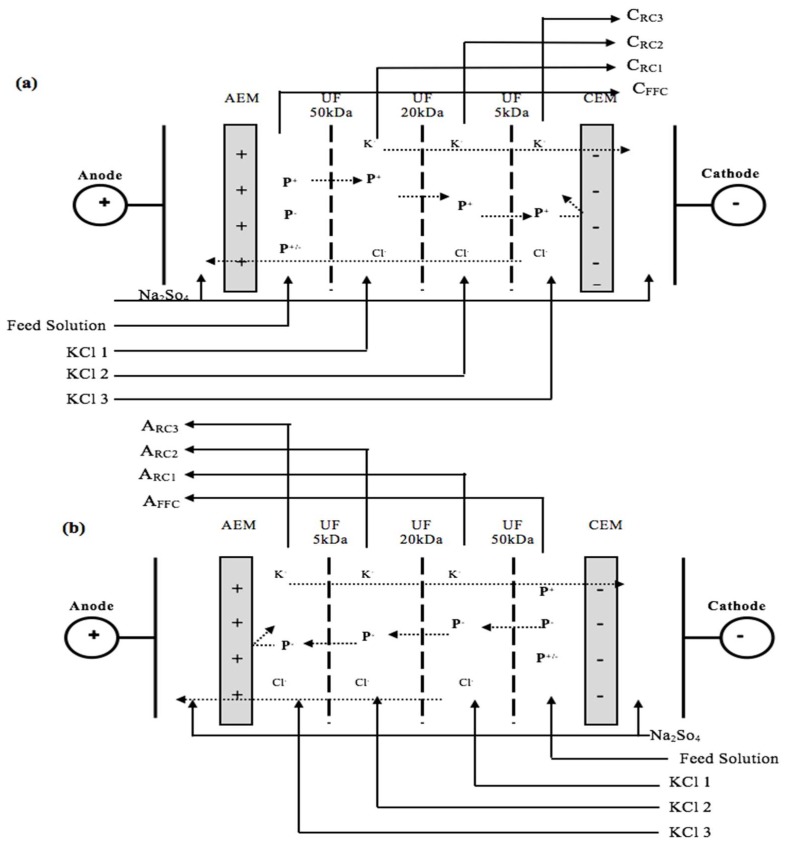
Layout showing EDUF membrane configurations, (**a**) cationic and (**b**) anionic, for the fractionation of the feed solution, which was an Unfractionated Salmon Protein Hydrolysate (U_SPH_). AEM: anion-exchange membrane, CEM: cation exchange membrane, UF membrane: ultrafiltration membrane, P^+^: cationic peptides; P^-^: anionic peptides, P^+/−^: neutral peptides, A_RC_: anionic recovery compartments, and C_RC_: cationic recovery compartments.

The electrodialysis cell used for the experiment was an MP type cell manufactured by ElectroCell Systems AB Company (Täby, Sweden). The cell had an effective surface area of 200 cm^2^ and was composed by one anion-exchange membrane (AEM), one cation-exchange membranes (CEM), and three UF membranes with MWCO of 50, 20, and 5 kDa (UF-50 kDa, UF-20 kDa, UF-5 kDa, respectively) as illustrated in Figure 4. The electrodes used were a dimensionally-stable anode (DSA) and a 316 stainless steel cathode. The electrical potential for the electrodialysis with ultrafiltration membrane (EDUF instead of EDFM since the filtration membrane was a UF membrane) was supplied by a variable 0–100 V power source. One polypropylene spacer (0.74 mm) was stacked in each compartment to promote turbulence. Two different cell configurations allowing the separation of cationic or anionic charged peptides from salmon protein hydrolysate were tested in this study:

For both configurations, the cell was composed of five closed loops: three of them contained 1.5 L of a KCl solution (2 g/L) for the recovery compartments, one loop contained the feed compartment, and the last one contained the electrode rinsing solution (20 g/L Na_2_SO_4_, 3 L), and was split in half between the anode and the cathode compartments. The solutions were circulated using five centrifugal pumps, and the flow rates were set at 2 L/min using flow meters (the electrode rinsing solution was maintained at 4 L/min and split in half between the anode and the cathode compartments) (Blue-White Industries Ltd., Huntington Beach, CA, USA).

Cationic configuration—The first EDUF cell configuration, shown in Figure 4a, was for the separation of cationic peptides. The UF membranes were placed in the cell according to their exclusion limits starting from the anode side to allow the migration of cationic peptides on the basis of their size and charge. The compartment containing a KCl solution circulating between the UF-50 kDa and UF-20 kDa was named the cationic recovery compartment 1 (C_RC1_). The cationic recovery compartment 2 (C_RC2_) was located between the UF-20 kDa and UF-5 kDa, and the cationic recovery compartment 3 (C_RC3_) between the UF-5 kDa and the CEM. The feed solution consisting of salmon protein hydrolysate (SPH, 1.5 L, 0.7% *w*/*v*) was circulated in the compartment between the UF-50 kDa and the AEM.

Anionic configuration—In this second configuration (Figure 4b), the UF membranes were arranged according to their MWCOs starting from the anode side to allow the migration of anionic peptides on the basis of their size and charge. The compartment containing a KCl solution circulating between the UF-50 kDa and UF-20 kDa membranes was called the “anionic recovery compartment 1” (A_RC1_), anionic recovery compartment 2 (A_RC2_) was located between the UF-20 kDa and UF-5 kDa membranes and finally the anionic recovery compartment 3 (A_RC3_) between the UF-5 kDa and AEM. The feed solution (SPH) was circulated in the compartment between the UF-50 kDa and the CEM.

#### 3.1.2. Electroseparation Protocol

The EDUF separations were performed according to the previous study of Roblet et al. (2016) [30]. Briefly, the EDUF separations were performed in batches for both cell configurations using a constant electrical field strength of 6 V/cm (corresponding to a current density varying between 0.005 and 0.008 A/cm^2^ during the treatment), for 6 h, at a controlled temperature (~16 °C) [34]. The SPH was diluted with demineralized water to obtain a final protein concentration of 0.7% (*w*/*v*). Following the results obtained by Roblet et al. [30], the pH of the SPH and recovery (KCl) solutions were adjusted to pH 6 before each run with 0.1 N NaOH and/or 0.1 N HCl and maintained constant thereafter. For each treatment, 5 mL of SPH and recovery solutions were collected every hour for further analysis. The electrical conductivity of the feed solution and recovery solutions was maintained at a constant level by adding KCl, following the recommendations of Suwal et al. (2015) [34]. Three replicates of each condition were performed. Finally, a CIP (cleaning-in-place) was performed at the end of each replicate according to the following process: 10 min with an acid solution (HCL 0.1 N), 20 min with a basic solution (NaOH 0.1 N), and finally 10 min with an acid solution (HCL 0.1 N). Then, the system was rinsed with distilled water until reaching a pH of 6.

### 3.2. Materials

#### 3.2.1. Hydrolysate Preparation

Salmon protein hydrolysate (SPH) was produced according to the procedure described previously by Jin, (2012) [40] and subsequently used by Chevrier et al., (2015) [6] and Roblet et al., (2016) [30]. Briefly, salmon frames were offered by Cooke Aquaculture. They were thawed, mechanically deboned, and homogenized in a 1.0 M NaOH solution. The proteins were isoelectrically precipitated at a pH of 4.5. Then, the proteins were first hydrolyzed with pepsin, and then by a mix of trypsin/chymotrypsin. Once hydrolysis was complete, the supernatant was filtered through a 5 µm pore size paper filter to remove insoluble molecules. Finally, the filtrate was ultrafiltered using a Prep/Scale Tangential Flow Filtration (TFF) 2.5 ft² cartridge with a molecular weight cut-off of 1 kDa (Millipore Corporation, Bedford, MA, USA). Permeates were collected, demineralized by conventional electrodialysis, and finally freeze-dried.

#### 3.2.2. Chemicals

KCl was obtained from ACP Inc (Montreal, QC, Canada) and Na_2_SO_4_ from Laboratoire MAT (Québec city, QC, Canada). Formic acid, 1.0 M HCl, and 1.0 M NaOH solutions were from Fisher Scientific (Montreal, QC, Canada), trifluoroacetic acid was purchased from J.T. Baker (Phillipsburg, NJ, USA). NaCl, Acetonitrile optima^®^ liquid chromatography-mass spectrometry (LC/MS), and water grade were from VWR international (Montréal, QC, Canada). Concerning the glucose uptake experiments, the alpha-Minimal Essential Medium (α-MEM), Fetal Bovine Serum (FBS), and trypsin (0,25% solution) were obtained from Invitrogen (Burlington, ON, Canada). The 2-déoxy-D-glucose (non-radioactive), CaCl_2_, Hepes-Na, and MgSO4 were purchased from Sigma Aldrich (Oakville, ON, Canada). D-2-deoxy-[^3^H] glucose was from Perkin Elmer (Woodbridge, ON, Canada) and Pierce^®^ BCA Protein Assay Kit BCA was from Pierce Biotechnology (Rockford, IL, USA). Insulin was from CHUL’s pharmacy (Québec, QC, Canada). Also, the L6 skeletal muscle cells line, derived from neonatal rat thigh skeletal muscle, were provided by Dr. A. Klip, Hospital for Sick Children (Toronto, ON, Canada).

#### 3.2.3. Membranes

Three UF membranes made of polyether sulfone (PES) with molecular weight exclusion limits or molecular weight cut-off (MWCO) of 50, 20, and 5k Da were purchased from Synder filtration (Vacaville, CA, USA). Unlike in classical filtration processes where higher pressure is applied, previous papers published on EDMF demonstrated that the MWCO of UF membranes should be about ten times higher than the size of proteins or peptides to be successfully migrated due to steric hindrance from the hydration layer [41]. Indeed, in an electro-ultrafiltration module, Bargeman et al. (2002) observed that the migration of α_S2_ casein f(183–207) was strongly reduced when a membrane with a MWCO of 20 kDa (six-times higher than the molecular weight of the peptide) was used due to the friction of peptides in the membrane pores [41]. This was also confirmed for EDUF by previous works by our team on peptides and chitosan oligomers [42]. While food-grade Neosepta CMX-SB cationic membranes and Neosepta AMX-SB anionic membranes were obtained from Tokuyama Soda Ltd. (Tokyo, Japan).

### 3.3. Analyses

#### 3.3.1. pH

The pH of all solutions was measured and kept constant throughout the experiments using a pH-meter model SP20 (Thermo Orion, West Chester, PA, USA) equipped with a VWR Symphony epoxy gel combination pH electrode (VWR, Montreal, QC, Canada).

#### 3.3.2. Relative Energy Consumption of the EDUF Process

The energy consumption during the EDUF process was calculated using Equation (1):

(1) $$EC=\int_{t=0h}^{t=6h}I*U\;dt$$

where, *EC* is the energy (Wh), *I* the current intensity (A), and *U* the voltage (V). The relative energy consumption during EDUF treatment was then calculated by dividing the total energy by total grams of peptides obtained at the end of the treatment.

#### 3.3.3. Peptide Concentration and Nitrogen Concentration Determination

To follow the peptide migration during the EDUF separation, the peptide concentrations in all the solutions were determined using micro bicinchoninic acid (µBCA) protein assay reagents (Pierce, Rockford, IL, USA) using bovine serum albumin (BSA) as the standard protein. The microplate was incubated with a mix of 150 µL of the sample and 150 µL of the working reagent, at 37 ˚C for 2 h. Then, the microplate was cooled to room temperature and the absorbance was read at 562 nm on a microplate reader (Thermomax, Molecular devices, Sunnyvale, CA, USA).

Nitrogen concentrations were analysed in final lyophilized fractions using a LECO Model 601-500 FP528 apparatus (LECO corporation, St. Joseph, MI, USA). Samples of 0.150 g were analyzed in duplicate. The protein content was determined using the protein factor of 6.25 (% Nitrogen × 6.25). The instrument was previously calibrated with ethylenediaminetetraacetic acid (EDTA).

#### 3.3.4. Final Peptide Migration Rates

Final migration rates of peptides (MR) in recovery compartments were calculated using Equation (2):

(2) $$MR=\frac{F\times L}{t\times S}$$

where, F is the concentration at t time in g/mL, L is the volume of the final solution in mL, t is the duration for reaching F concentration in one hour, and S is the total UF membrane area in m^2^.

#### 3.3.5. Reverse Phase Ultra Performance Liquide Chromatography (RP-UPLC) and Tandem Mass Spectrometry (MS/MS) Analyses

The RP-UPLC analyses were done according to the previous study from Durand et al. (2019) [43]. Briefly, a 1290 Infinity II UPLC (Agilent Technologies, Santa Clara, CA, USA) was used to separate samples before entering the samples in the mass spectrometer. The EDUF fractions were diluted to 0.5 mg/mL, then filtered through 0.22 µm PVDF filters into a glass vial. Then, 5 µL of each sample were loaded onto an Acquity UPLC CSH 130Å, 1.7 µm C18 column (2.1 mm i.d.× 150mm, Waters Corporation, Milford, MA, USA) at a flow rate of 400 µL/min and a temperature of 45 °C. A linear gradient from 2% to 25% over 50 min and ramping to 90% over 57 min were used. The gradient consisted of a solvent A, which was LC-MS grade water with 0.1% formic acid, and a solvent B, which was LC-MS grade ACN with 0.1% formic acid. Each sample was run in triplicate for statistical evaluation of technical reproducibility.

A hybrid ion mobility quadrupole TOF mass spectrometer (6560 high definition mass spectrometry (IM-Q-TOF), Agilent, Santa Clara, USA) was used to identify the composition of each EDUF fraction. All LC-MS/MS experiments were acquired using Q-TOF. Signals were recorded in positive mode at Extended Dynamic Range, 2 GHz, 3200 *m*/*z*, with a scan range between 100 to 2000 m/z. Nitrogen was used as the drying gas at 13.0 L/min and 150 °C, and as a nebulizer gas at 30 psig. The capillary voltage was set at 3500 V, the nozzle voltage at 300 V, and the fragmentor at 400 V. Data analyses were done using the Agilent Mass Hunter Software package (LC/MS Data Acquisition, Version B.07.00 and Qualitative Analysis for IM-MS, Version B.07.00 with BioConfirm Software, Agilent, Santa Clara, CA, USA). An additional search was done using the Spectrum Mill MS Proteomics Workbench Rev B.05.00.180. The Salmo salar protein database [36] was used to search for and identify potential peptides.

#### 3.3.6. Glucose Uptake Experiments

Glucose uptake experiments were conducted as previously described by Roblet et al., (2016) [30]. L6 skeletal muscle cells were grown in an α-minimum essential medium (α-MEM) containing 2% (*v*/*v*) fetal bovine serum (FBS) in an atmosphere of 5% CO_2_ at 37 °C [44]. Cells were plated at 600,000 cells/plate in 24-well plates to obtain about 25,000 cells/mL. The cells were incubated for 7 days to reach their complete differentiation to myotubes (7 days post-plating). L6 myotubes were deprived of FBS for 3 h, with a α-MEM containing 0% of FBS. Then, the cells were incubated for 75 min, with 10 µL of EDUF fractions at a concentration of 1 µg/mL and 1 ng/mL. Finally, insulin was added (10 µL at 1.10–5 M) for 45 min. Experiments were repeated nine times, and each repetition was run in triplicate. After experimental treatments, cells were rinsed once with 37 °C HEPES-buffered solution (20 mM HEPES, pH 7.4, 140 mM NaCl, 5 mM KCl, 2.5 mMMgSO_4_, and 1 mMCaCl_2_) and were subsequently incubated in HEPES-buffered solution containing 10 µM2-deoxyglucose and 0.3 µCi/mL2-deoxy-[^3^H] glucose for 8 min. Then, the cells were rinsed three times with 0.9% NaCl solution at 4 °C and then frozen. The next day, the cells were disrupted by adding 500 µl of a 50 mM NaOH solution. The radioactivity was determined by scintillation counting. Protein concentrations were determined by the BCA method, and results of glucose uptake were expressed as relative value over the vehicle, which was the control.

#### 3.3.7. Statistical Analyses

Peptide concentration, relative abundance, membrane conductivity and thickness, and glucose-transport array values between different peptide fractions were subjected to a one-way analysis of variance (Anova) using SAS software version 9.1 (SAS institute Inc., Cary, NC, USA) with a significant *p* values of 0.05 for acceptance. Duncan and Dunnett post-hoc tests were used.

The relative energy consumption was compared by student’s *t*-test (*p* < 0.05 as probability level for acceptance).

## 4. Conclusions

The simultaneous separation of peptides by three UF membranes (50, 20, and 5 kDa MWCO) stacked in an electrodialysis system allowed for the generation of specific cationic and anionic fractions with different MW profiles and levels of glucose uptake response. As expected, significant decreases were observed concerning the peptide concentrations in the recovery compartments in the order of C_FFC_ > C_RC1_ > C_RC2_ > C_RC3_ and A_FFC_ >A_RC1_ >A_RC2_ > A_RC3_ for the cationic and anionic configurations, respectively. Moreover, the peptide profiles in terms of MWs followed the same tendency as the peptide concentrations with HMWPs concentrated in the feed compartment while LMWPs were able to cross the three UF membranes stacked in the electrodialysis and some reached the last compartment. For the first time, a triple size-separation by EDUF allowed for the concentration, in one step, of bioactive peptides in the C_RC1_ and inhibitor peptides in C_CRC3_. Coupling the EDMF-based separation of peptides with bioassay-guided validation of their metabolic activity with LC-MS identification allowed for the identification of eleven potential antidiabetic peptides from a complex salmon frame protein hydrolysate containing more than 250 different peptides. Hence, a pre-separation by EDUF appears to be a new powerful tool and key step for accelerating peptide identification. Nevertheless, further mass spectrometry analysis is needed to identify and determine the distribution of each peptide in the fractions and if the bioactivity is linked to one or more peptides in those fractions. These peptides were recently synthesised and their bioactivity measurements during in-vitro tests, alone or in combination, are currently under way to confirm the anti-diabetic activity of these peptides.

## Figures and Tables

**Table 1:** The relative energy consumption, peptide migration rate, and peptide concentration in cationic and anionic configuration compartments. Lowercase letters are used to compare the three recovered compartments of the same configuration, means with different lowercase letters are significantly different (*p* < 0.05). Whereas capital letters are used to compare the recovered compartments between anionic and cationic configuration, means with different capital letters are significantly different (*p* < 0.05).

EDUF Fractions	Peptide (%)	Peptide Migration Rate (g/m^2^·h)	Relative Energy Consumption (Wh/g)
Unfractionated salmon protein hydrolysate (U_SPH_)	80.83 ± 2.14	—	—
**EDUF Configuration a**	—	—	512.56 ± 95.59 ^b^
Cationic Final Feed Compartment (C_FFC_)	67.16 ± 1.72	—	—
Cationic Recovery Compartment 1 (C_RC1_)	9.20 ± 2.29	3.19 ± 0.14 ^a,A^	—
Cationic Recovery Compartment 2 (C_RC2_)	1.79 ± 0.54	0.73 ± 0.06 ^b,A^	—
Cationic Recovery Compartment 3 (C_RC3_)	0.33 ± 0.19	0.13 ± 0.06 ^c,A^	—
**EDUF Configuration b**	—	—	849.71 ± 80.18 ^a^
Anionic Final Feed Compartment (A_FFC_)	71.88 ± 0.94	—	—
Anionic Recovery Compartment 1 (A_RC1_)	6.67 ± 0.7	2.24 ± 0.21 ^a,B^	—
Anionic Recovery Compartment 2 (A_RC2_)	0.37 ± 0.14	0.21 ± 0.06 ^b,B^	—
Anionic Recovery Compartment 3 (A_RC3_)	0.15 ± 0.008	0.01 ± 0.04 ^c,B^	—

**Table 2:** Cationic and anionic peptides simultaneously present in each cationic (C_FFC_, C_RC1_, and C_RC3_) and anionic (A_RC1_, A_RC2_, and A_RC3_) bioactive fraction.

	Cationic Peptide’s				Anionic Peptide’s		
#	Retention Time (min)	Molecular Weight (Da)	Frequency *	#	Retention Time (min)	Molecular Weight (Da)	Frequency *
1	6.655	627.3711	8	1	9.878	416.2344	8
2	8.844	671.3281	8	2	10.172	456.2654	8
3	12.663	794.4654	9	3	14.991	531.2895	9
4	13.862	507.2681	9	4	15.240	409.1843	9
5	13.910	843.4589	9	5	15.271	503.2657	8
6	13.910	719.4219	9	6	15.750	502.2628	9
7	13.915	956.5451	9	7	16.536	444.2577	9
8	14.035	1085.6240	8	8	18.710	869.5485	9
9	14.141	801.4025	8	10	19.019	502.2713	8
10	16.378	805.4078	9	11	19.783	407.2053	9
11	16.491	372.2368	8	12	20.905	515.3020	8
12	18.962	473.3213	9	13	21.399	407.2056	9
13	18.963	643.4267	9	14	22.346	458.2737	9
14	21.274	634.3794	9	15	23.048	494.2369	9
15	25.261	409.2029	9	16	23.091	542.2369	9
16	27.316	434.2523	9	17	24.073	431.2728	9
17	30.407	1014.5737	9	18	24.089	592.2850	9
				19	26.698	829.3969	9
				20	26.855	458.2721	9
				21	29.435	660.3509	8

* For each configuration, nine fractions were compared (three compartments in triplicate). A frequency of nine means that the molecular mas was found in each compartment and for each repetition.
